# Severe cartilage damage from a broken absorbable screw head after fixation of an avulsion fracture of the tibial attachment of the posterior cruciate ligament: A case report

**DOI:** 10.1097/MD.0000000000005180

**Published:** 2016-10-28

**Authors:** Qiangqiang Li, Kai Song, Ye Sun, Haojun Zhang, Dongyang Chen, Qing Jiang

**Affiliations:** a Department of Sports Medicine and Adult Reconstructive Surgery, Nanjing Drum Tower Hospital, Clinical College of Nanjing Medical University; b Laboratory for Bone and Joint Diseases, Model Animal Research Center, Nanjing University; c Department of Sports Medicine and Adult Reconstructive Surgery, Nanjing Drum Tower Hospital Affiliated with the Medical School of Nanjing University, Nanjing, Jiangsu, PR China.

**Keywords:** absorbable screw, severe chondral damage, tibial avulsion fracture of the PCL

## Abstract

**Introduction:**

The use of bioabsorbable interference screws has become popular for treatment of avulsion fractures of the posterior cruciate ligament (PCL). Complications are uncommon. We report a case of severe chondral damage caused by the early breakage of an absorbable screw head after fixation of an avulsion fracture of the tibial attachment of the PCL. The patient felt a sudden locking of the knee when getting off a car at 4 months after the PCL surgery. MRI revealed intraarticular migration of the head of the interference screw. During revision surgery, the broken part was removed without incident, and severe cartilage damage was observed. The patient experienced a complete resolution of symptoms at the 6-month follow-up.

**Conclusion:**

MRI examination is recommended in case of sudden locking of the knee for patients undergoing PCL fixation with bioabsorbable interference screws. Surgical treatment should be performed immediately when screw breakage was confirmed.

## 1 Introduction

The use of bioabsorbable interference screws has become a standard procedure for fixation of avulsion fractures of the posterior cruciate ligament (PCL).^[^1–4^]^ This approach has become popular due to good fixation,^[^2 3^]^ avoidance of reoperation,^[3]^ and improved postoperative MRI findings. Meanwhile, complications related with the application of bioabsorbable interference screws are uncommon.

We report a case of severe chondral damage caused by the early breakage of an absorbable screw head after PCL surgery. Prior publications have only reported the breakage of bioscrews in cases of anterior cruciate ligament (ACL) and PCL reconstruction.^[^5–9^]^ This case presents the incidence of screw breakage, intraarticular migration of the fragment, and severe chondral damage.

## 2 Case report

A 52-year-old man with an avulsion fracture of the tibial attachment of the PCL underwent posterior middle mini-incision fixation with a poly-l-lactic acid (PLLA) absorbable screw in a primary hospital. After surgery, the patient followed a standard rehabilitation protocol with restrictive motion, partial weight bearing and bracing for 6 weeks.

**Figure 1:**
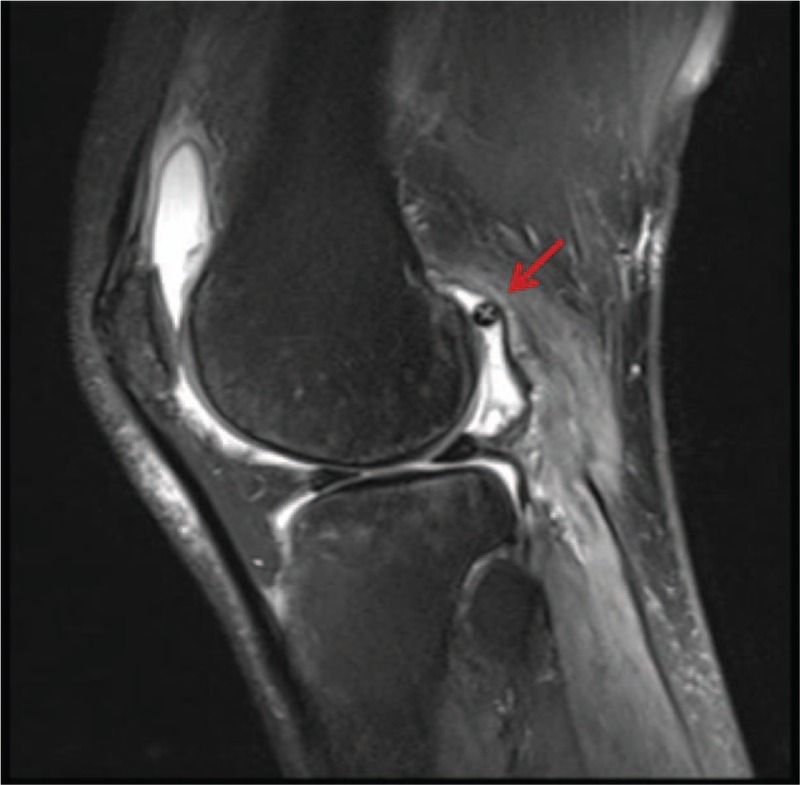
MRI 5 months after the initial operation revealing osteochondral lesions on the tibial and femoral sides and the head of the interference screw lying in the back of the joint capsule compartment (red arrows).

Eight weeks after surgery, the patient was satisfied with the surgical outcome, as he was able to have a flexion/extension 140°-0°-0° range of motion with no signs of instability or locking. Moreover, he was able to participate in his previous level of sporting activity with no complications. However, at the 4th month after surgery, the patient reported a sudden pain in the operated knee with locking and swelling after the knee was twisted when getting off a car. Subsequently, the patient repeatedly experienced the sensation of locking. MRI did not reveal any osteochondral lesions except for a foreign body that appeared similar to the head of the interference screw, which was found lying in the back of the joint capsule compartment (Fig. 1). The patient refused to take any treatment until he came to our hospital 2 months later.

Physical examination showed that the patient's range of flexion/extension motion had been reduced from 140°-0°-0° to 80°-0°-0°. There was no sign of muscle wasting, and the surgical scars had healed without signs of infection. Patellar and collateral ligament examinations were unremarkable. Palpation revealed tenderness over the lateral joint line. Lachman and posterior drawer tests were grade 1 positive with a solid endpoint. Pivot shift was negative. Based on the initial diagnosis, we suspected a broken absorbable screw head could exist as a loose intraarticular body after the initial operation.

**Figure 2:**
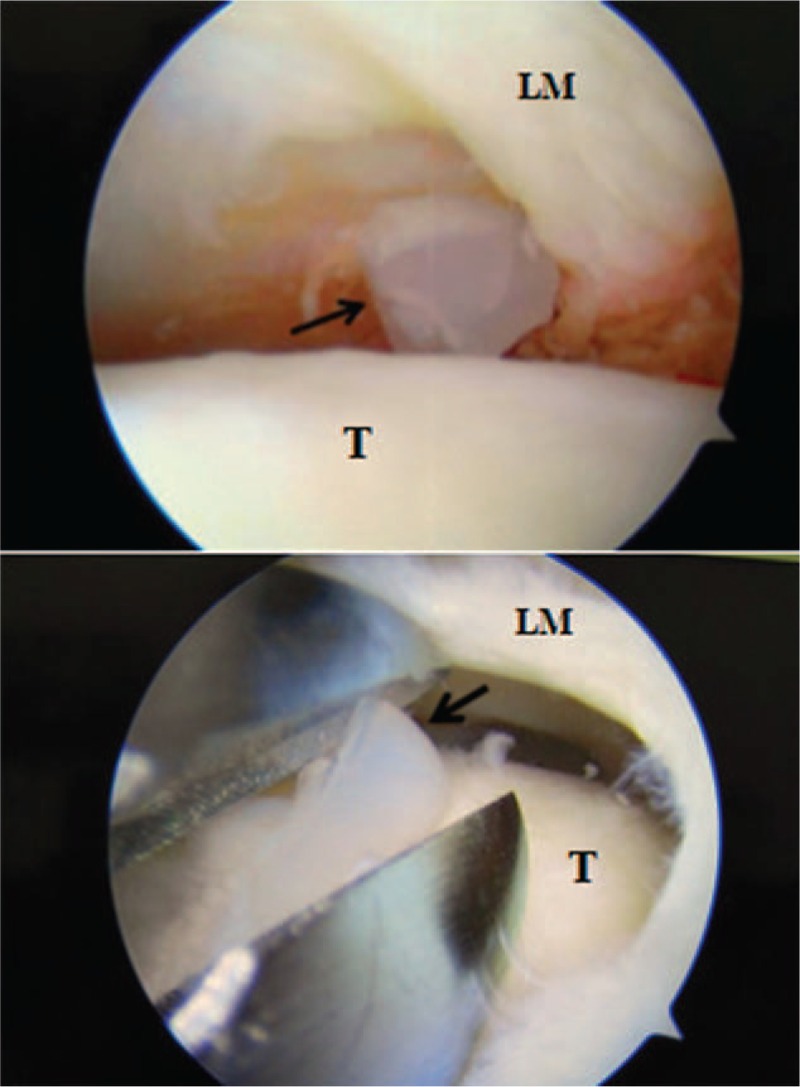
Three pieces of the head of the screw were found lying loose in the posterior joint capsule compartment (black arrows) during arthroscopy. LM = lateral meniscus, T = lateral tibial plateau.

**Figure 3:**
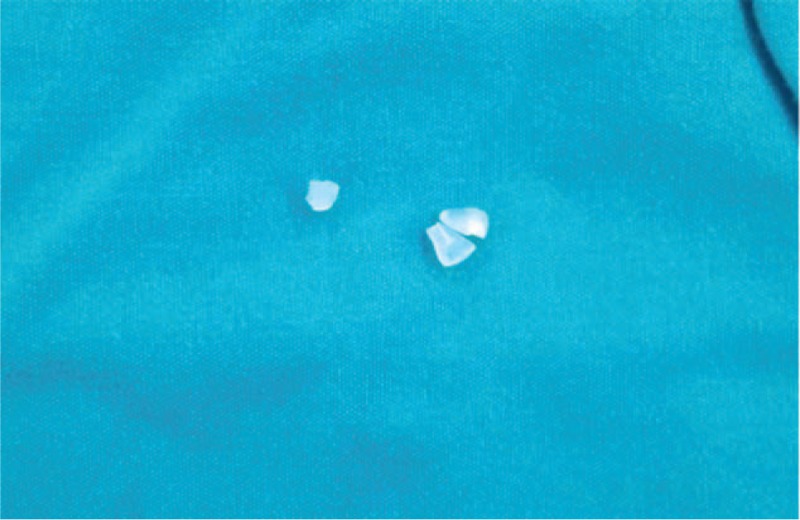
Remnants of the screws after its removal from the joint.

**Figure 4:**
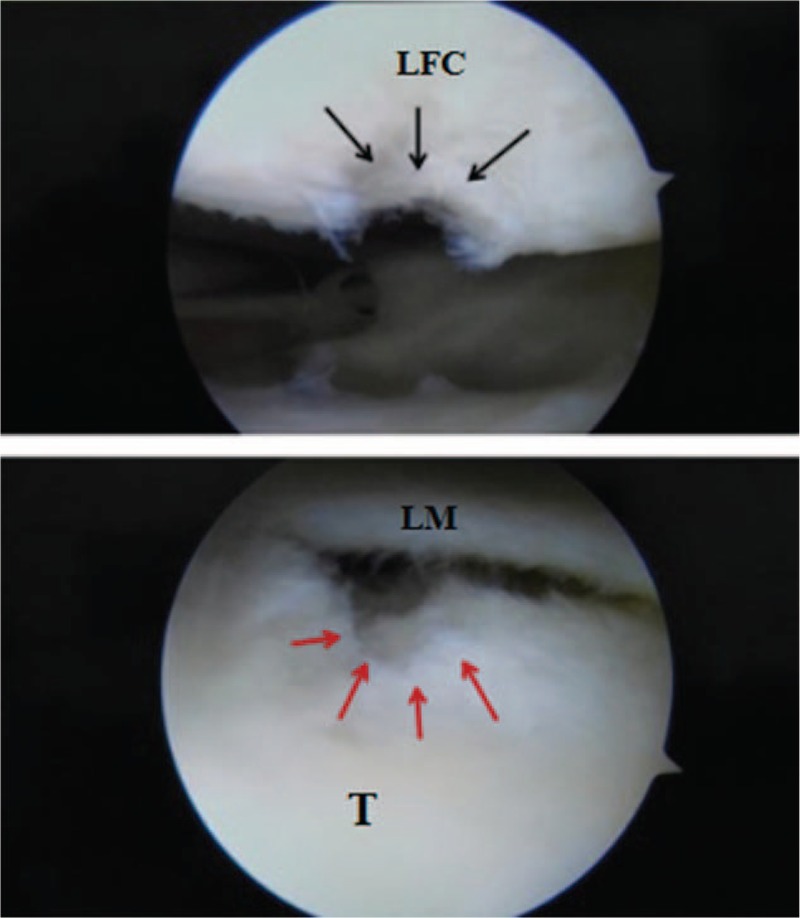
Arthroscopy revealing articular cartilage that indicates an ICRS III ulcer on the femoral side (black arrows) and an ICRS IV ulcer on the tibial side (red arrows). LFC = lateral femoral condyle, LM = lateral meniscus, T = lateral tibial plateau.

During revision arthroscopy, no signs of injury to the meniscus were observed, and the PCL was intact. The head of the interference screw was found to have broken into 3 pieces in the posterior joint capsule compartment (Fig. 2). These pieces were removed without any complications (Fig. 3). We were impressed by the severe chondral damage to the lateral femoral condyle (ICRS grade III) and to the lateral tibial plateau (ICRS grade IV) (Fig. 4).

After removal of the screw, the patient had a flexion/extension 145°-0°-0° range of motion and experienced a complete resolution of symptoms at his 6-month follow-up.

## 3 Discussion

In knee surgery, bioabsorbable interference screws are being widely used for ACL and PCL reconstruction and for fixation of avulsion fractures of the tibial attachment of the PCL. Six cases of the intraarticular migration of absorbable tibial interference screws have been described to date.^[^5–9^]^ Lembeck and Wülker ^[5]^ presented a case of severe chondral damage caused by late breakage of the screw after ACL reconstruction, which was similar with the case in this study. To our knowledge, this case study is the first article to report severe cartilage damage resulted from a broken absorbable screw head after fixation of a tibial avulsion fracture of the PCL.

**Figure 5:**
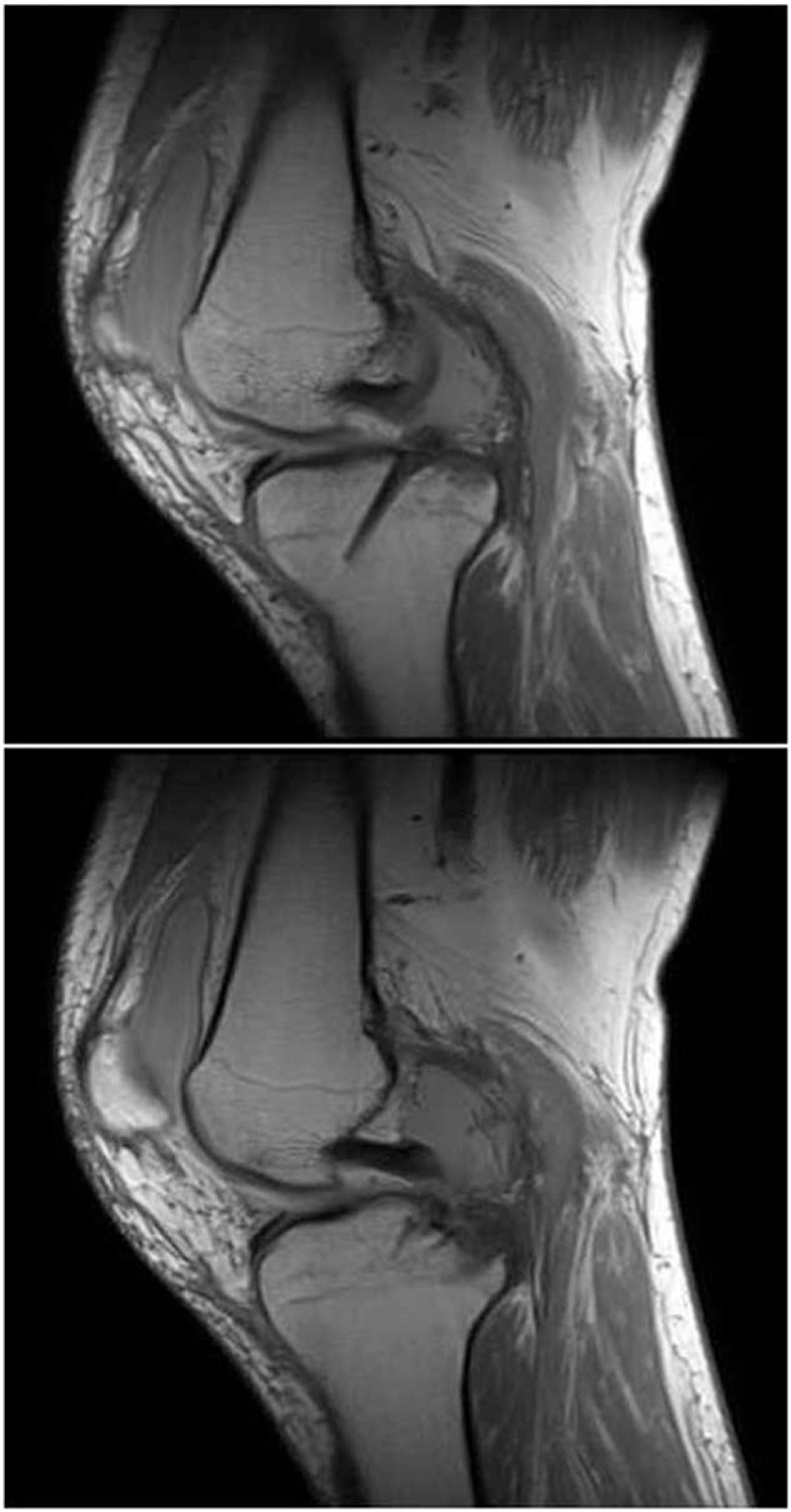
MRI 5 months after the initial operation revealing that the position of the absorbable screw was more anterior and medial than normal.

Based on preoperative MRI, we speculated that the breakage of the screw was attributed to the position of the screw, which was more anterior and medial than normal (Fig. 5). Poor learning curve of the prior surgeons may lead to the nonanatomical fixation. The angle between the screws drilled tunnel and the tibial plateau was inappropriately large, thus leading to altered stress concentration at the tibial attachment of the PCL. We assumed that during the postoperative rehabilitation, there was continuous force on the screw due to the abnormal stress, and the screw eventually broke when a knee sprain occurred.

Previous in vitro tests have revealed that PLLA screws could lose 50% of their compression strength due to hydrolytic degradation between 2 and 5 months after surgery.^[10]^ During revision arthroscopy of this patient, although we detected that the hardness of the broken absorbable screw was similar to normal, we still could not exclude the possibility of screw degeneration. Arthroscopy revealed an ICRS IV ulcer on the tibial side and an ICRS III ulcer on the femoral side of the patient's articular cartilage, which were not demonstrated by the preoperative MRI. Herein we speculated that the broken screw existed as a foreign body in the joint cavity between the tibia and the femoral condylar platform, subsequently leading to severe cartilage damage.

Recently, increasing numbers of published reports have addressed PCL avulsion fractures and the related modality of treatment. The stable fixation of the avulsed tibial attachment of the PCL and good functional outcomes can be achieved either by arthroscopically assisted methods^[^1–4^]^ or by open exposure.^[^11–14^]^ However, there remains no consensus regarding the treatment of this type of injury.^[15]^ The choice of operation method has typically been based on the size of the avulsed fragment and the choice of the internal fixation material. Specifically, surgical reconstruction is recommended for even minimally displaced fragments.^[16]^

At present, the main fixation materials are fixed sutures, steel wires, and screws.^[^3 13 17–19^]^ The use of bioabsorbable screws has been increasing dramatically in recent years due to unique advantages of this approach, such as the avoidance of reoperation and good fixation. An initial concern with bioabsorbable screws was their inferior mechanical strength compared with metallic implants.^[20]^ However, biomechanical tests of these screws have revealed sufficient pull-out strength,^[21]^ and reports have indicated that bioabsorbable interference screws made of polylactic acid have an advantage over those made of surgical steel or titanium, including less laceration of the graft, no interference with MRI examination, and no interference with subsequent knee surgery.^[9]^

Complications associated with the use of bioabsorbable screws are uncommon. The most frequent complication is the screw breakage during implantation,^[22]^ which can result in severe cartilage damage or meniscus injury. The key points for a successful implantation include an oblique drilling tunnel, fixed strength, and sufficient drilling length. In addition, it is recommended to use a screw tap before tapping and employ a screw length greater than the depth of tapping.

This report demonstrates that the early breakage of bioabsorbable interference screws can result in severe chondral damage. Surgeons should be aware of the screw breakage when applying bioabsorbable interference screws to the treatment of intraarticular fracture. For patients having received PCL fixation with bioabsorbable interference screws, MRI examination is recommended in case of sudden locking of the knee, which can be indicative of the screw breakage. For patients with a diagnosed screw breakage, surgical treatment should be performed immediately in case of further damage.

## Acknowledgment

The authors are grateful to all study participants.
